# Who are the missing men? Characterising men who never tested for HIV from population‐based surveys in six sub‐Saharan African countries

**DOI:** 10.1002/jia2.25398

**Published:** 2019-10-20

**Authors:** Caitlin Quinn, Damazo T Kadengye, Cheryl C Johnson, Rachel Baggaley, Shona Dalal

**Affiliations:** ^1^ Department of HIV/AIDS World Health Organization Geneva Switzerland; ^2^ African Population and Health Research Center Nairobi Kenya

**Keywords:** HIV/AIDS, men, HIV testing, Africa South of the Sahara, Health surveys

## Abstract

**Introduction:**

We sought to characterize men who had never tested for HIV, understand factors associated with not testing, and measure survey HIV test uptake among never testers. We analysed nationally representative Demographic and Health Surveys of six African countries from 2013 to 2016: Ethiopia, Malawi, Zimbabwe, Rwanda, Lesotho and Zambia.

**Methods:**

Eligible men were household residents or overnight visitors aged 15 to 59 years. We analysed questionnaire responses on HIV testing, known behavioural risk factors, and corresponding HIV laboratory results. We used survey‐weighted logistic regression to identify factors associated with never testing for HIV.

**Results:**

Approximately double the proportion of men had never tested for HIV compared to women (Malawi: 30% vs. 17%, *p *<* *0.0001; Zimbabwe: 35% vs. 19%, *p *<* *0.0001; Lesotho: 34% vs. 15%, *p *<* *0.0001; Zambia: 36% vs. 20%, *p *<* *0.0001); although, less of a differential existed in Ethiopia (54% vs. 56%, *p *=* *0.12) and Rwanda (19% vs. 14%, *p *<* *0.0001). When offered a test during the survey, 85% to 99% of sexually active men who reported never previously testing, accepted testing. HIV positivity ranged from <0.05% to 14% for never tested men. After adjusting for age, factors associated with never having tested for HIV were never being married (aOR range: 1.46 to 10.39), not having children (aOR: 1.36 to 3.59) and lower education (less than primary education aOR: 2.77 to 5.59).

**Conclusions:**

Although higher proportions of men than women had never tested for HIV, 85% to 99% of men did accept a test when offered. Finding opportunities to offer HIV testing to single men without children, older men who have never tested, and those disadvantaged with less schooling and employment, alongside other facility and community‐based services, will be important in identifying those living with undiagnosed HIV and improving men's health.

## 1 Introduction

Reaching people with undiagnosed HIV is critical to curbing the HIV epidemic. The first 90 of the United Nations 90‐90‐90 targets is for 90% of people with HIV to know their HIV status by 2020. In 2017, approximately 75% of people with HIV globally were aware of their status, yet gaps across the 90‐90‐90 continuum were greater for men 1. Globally, men experience shorter lifespans, greater threats to health and safety, and less access to health care than women 1, 2. According to recent estimates, HIV‐related mortality is higher among men compared to women in sub‐Saharan Africa. Such trends are largely due to lower testing coverage and late presentation for HIV testing and treatment 1. Greater emphasis on reaching men with HIV testing is needed to improve men's health and well‐being, particularly in sub‐Saharan Africa, the region most affected by the HIV epidemic 3.

As men often test later for HIV 4, 5, 6, 7, they link to care at later stages of disease 5, 8, and in some settings have been shown to experience nearly twice the mortality rate of women 7, 9, 10. Yet, recent trials in Zambia 11 and South Africa 12 and a retrospective cohort study in Nigeria 13 have shown that after uptake of testing, men and women have similar rates of antiretroviral treatment (ART) initiation and long‐term retention. Thus, earlier HIV testing of men is critical for earlier linkage to life‐saving treatment and preventing onward transmission to sexual partners. Data from South Africa shows a decline in HIV incidence for men, but not for women, indicating an urgent need for undiagnosed men to be tested, linked to ART and achieve viral suppression to realize epidemic control 14.

A systematic review found that “fear of getting the disease” and perceived low risk prevented men from receiving screening for a wide variety of health conditions 15. A longitudinal qualitative study in East Africa found that men's lower participation in HIV testing is influenced by highly mobile labour opportunities and certain male gender norms which dissuade care‐seeking 16. Although it is broadly understood that men continue to lag behind women in utilizing clinic‐based HIV testing services (HTS), opportunities for men to test are not readily available. HTS has been routinely provided for adult women in sexual and reproductive health (SRH) services, specifically during antenatal care (ANC). In most high burden settings, routine testing has been provided in ANC since the early 2000s with high uptake, leading to greater testing coverage for women 17. Men do not routinely attend health services where HIV testing is offered and have fewer opportunities for testing in clinical settings. Men are also less likely than women to be present at home and therefore do not benefit from home‐based testing services 12.

Identifying the characteristics of men who do not go for HIV testing is needed to plan and implement testing services which can better reach them. Determinants of HIV testing among men has been studied in several reports 18, 19, 20, 21, 22, 23, 24, 25, including analyses of Demographic and Health Surveys (DHS) 19, 20, 22, 23. Existing evidence shows that men who were younger 24, 25, had less education 18, 20, 24, 25, did not have an occupation 18, had stigmatising views of HIV 21, 22 and who lacked HIV knowledge 22 were more likely to report never testing for HIV. In this study, we sought to characterize men who had never tested for HIV in sub‐Saharan Africa, understand the factors associated with not testing, and measure men's uptake and positivity rates when offered HIV testing in a survey setting.

## 2 Methods

### 2.1 Study design

We used data from nationally representative Demographic and Health Surveys (DHS) conducted in six countries in sub‐Saharan Africa in 2013 or later: Ethiopia in 2016 26, Malawi in 2015 to 2016 27, Zimbabwe in 2015 28, Rwanda in 2014 to 2015 29, Lesotho in 2014 30 and Zambia in 2013 to 2014 31. These six countries were selected for the study because they had recent surveys (2013 to 2016) and contained linked HIV testing data for the analysis.

Eligibility criteria for the DHS and household sampling procedures are summarised here but additional detail is available elsewhere 26, 27, 28, 29, 30, 31, 32. Men were household residents or overnight visitors from all sampled households in Ethiopia, Zimbabwe and Zambia, every second household in Rwanda and Lesotho, and from every third household in Malawi. Age eligibility for men was 15 to 59 years in Ethiopia, Lesotho, Rwanda and Zambia; 15 to 54 years in Malawi and Zimbabwe. Survey data for women was also reviewed on HIV testing history in order to report a comparison with men on rates of ever testing for HIV.

All men in the surveys were offered HIV testing. Respondents were provided verbal counselling and printed support materials 33. Blood spot samples were collected via finger‐prick for voluntary HIV testing from those who consented. Test results were not returned to respondents who opted to undergo testing in any of the surveys analysed. We analysed self‐reported responses on sociodemographic information, HIV testing history and known behavioural risk factors from the individual men's questionnaires. We also used the biomarkers datasets to assess respondents’ corresponding laboratory HIV test results. HIV testing was conducted using the national testing algorithm for each country.

### 2.2 Ethics

All DHS surveys and data collection procedures were approved by the ICF Institutional Review Board (IRB) and an IRB in the host country where the research was carried out 33. Participation was voluntary and all individuals provided informed consent prior to the interview and HIV testing 33.

### 2.3 Data management and statistical analysis

In this analysis, we included survey participants who responded to the question on whether they had previously been tested for HIV. Participant characteristics and known HIV risk factors were assessed. We used corresponding laboratory results to determine the rate of acceptance of HIV testing in the survey and HIV serostatus.

The outcome variable of interest was having never tested for HIV. Having never tested for HIV was obtained from individual responses to the question: “Have you ever tested for HIV?” with responses coded 1 for Yes and 0 for No. In this study, we modelled never testing as a primary outcome of interest. The independent variables included in the analyses were age of respondent (continuous, 15 to 59 years), place of residence (urban, rural), highest level of education attained (none, primary, secondary, higher than secondary), marital status (never in union/living with partner, currently in union/living with partner, formerly in union/living with partner), employment status at time of survey (employed, not employed), wealth quintiles, has children (yes, no), travels away from home (yes, no), has health insurance (yes, no), is circumcised (yes, no) and lifetime sexual partners (1 only, 2 or more).

Data from each of the six countries were analysed independently. Survey results were weighted using country‐specific sampling weights 32. To determine associations between having never tested and several independent variables, we used survey‐weighted logistic regression for both bivariate and multivariate analyses, which takes the sample design into account and provides inferences for the entire study population. All variables of interest were included in the multivariate regression model. All tests were two‐tailed, and a value of *p *<* *0.05 was considered significant with 95% confidence intervals (CI). All statistical analyses were performed using STATA version 14 34.

## 3 Results

**Figure 1:**
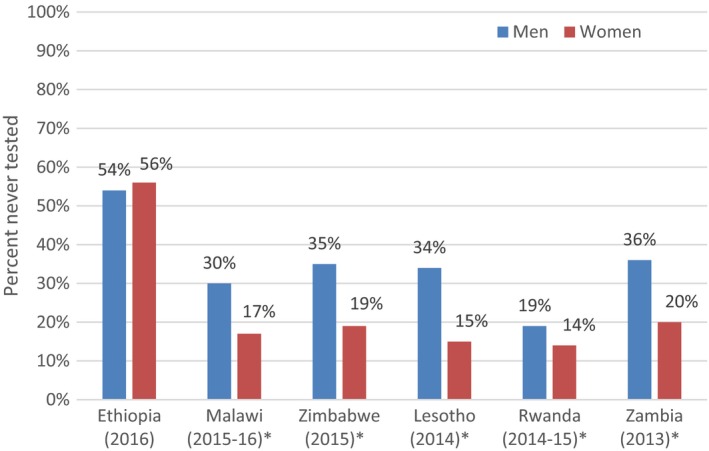
Proportion of men and women who had never been tested for HIV by country. *p <* *0.001

Approximately double the proportion of men had never tested for HIV compared to women in Malawi (30% of men never tested vs. 17% of women, *p *<* *0.0001), Zimbabwe (35% vs. 19%, *p *<* *0.0001), Lesotho (34% vs. 15%, *p *<* *0.0001) and Zambia (36% vs. 20%, *p *<* *0.0001); although, less of a differential existed in Ethiopia (54% vs. 56%, *p *=* *0.12) and Rwanda (19% vs. 14%, *p *<* *0.0001) (Figure 1.).

### 3.1 Characteristics of men who had never tested for HIV

Across most countries, men who had never tested for HIV were younger, lived predominantly in rural areas, had less education, were primarily never married and did not have children compared to men who had previously tested for HIV (Table S1). Although trends were broadly similar across countries, some differences were seen for specific variables. For example, most men who had never tested lived in rural areas (range: 56% to 88%), except in Rwanda where 16% lived in rural areas. Similarly, most men (range: 51% to 90%) who had never tested had primary education or less, except in Zimbabwe where 32% did. In all countries, men who never tested for HIV were largely never married (range: 53% to 85%) and did not have any children (range: 51% to 85%).

Compared with men who had tested for HIV, men who never tested reported higher unemployment (range: 24% to 39% vs. 9% to 27% of tested men), except in Ethiopia where both tested and never‐tested men reported lower unemployment (10% vs. 5%). Never‐tested men who were employed were less likely than tested employed men to be in professional services and more likely to be engaged in agricultural work in all countries. Compared to men who had tested previously, men who never tested were predominantly in the lower wealth quintiles in Ethiopia, Zimbabwe and Lesotho.

### 3.2 Risk factors for HIV among respondents who never tested and ever had sex

The majority of sexually experienced never‐tested men had sexual debut by age 19 (range: 46% to 83% across the six countries). Most never‐tested men (range: 49% to 58% across countries) reported two to five lifetime sex partners (highest in Lesotho: median 3 lifetime partners (IQR 2 to 7); lowest in Ethiopia: median 1 lifetime partner (IQR 1 to 3)). Men reported low condom usage at last sex with their most recent partner (range: 3% to 58% across countries). Between 2% and 22% of never tested men reported that they had paid for sex (2% in Ethiopia up to 22% in Zimbabwe). Most men reported being circumcised in Ethiopia (92%) and Lesotho (69%), but fewer were in Malawi (22%), Zimbabwe (6%), Rwanda (20%) and Zambia (17%). Men who had been tested for HIV were significantly more likely to be circumcised than never tested men in all countries apart from Ethiopia. Many African males may have been traditionally circumcised, though the survey data does not differentiate it from voluntary medical male circumcision (VMMC).

### 3.3 Factors associated with never testing for HIV among men

Across countries, factors associated with never having tested for HIV among men, after adjusting for age, were never being married (adjusted odds ratios for countries (aOR) ranging from 1.46 to 10.39), not having children (aOR 1.36 to 3.59) (except for in Ethiopia) and having primary education (aOR 2.34 to 3.49) or less than primary education (aOR 2.77 to 5.59) (Table 1).

**Table 1:** Factors associated with never testing for HIV among men

Variable	Ethiopia 2016 (N = 12,688)	Malawi 2015 to 2016 (N = 7478)	Zimbabwe 2015 (N = 8396)	Lesotho 2014 (N = 2931)	Rwanda 2014 to 2015 (N = 6217)	Zambia 2013 to 2014 (N = 14,765)
	aOR (95% CI)	aOR (95% CI)	aOR (95% CI)	aOR (95% CI)	aOR (95% CI)	aOR (95% CI)
Age (years)	1.01 (1.00, 1.02)	1.00 (0.99, 1.01)	1.01 (0.88, 1.17)	0.98** (0.96, 0.99)	1.06** (1.05, 1.08)	1.02** (1.01, 1.02)
Education						
No education	4.09** (2.94, 5.68)	5.59** (2.92, 10.70)	3.73** (1.89, 7.35)	3.66** (2.00, 6.72)	2.77* (1.36, 5.62)	3.81** (2.73, 5.33)
Primary	2.40** (1.78, 3.22)	3.26** (1.80, 5.93)	3.49** (2.56, 4.76)	2.68** (1.59, 4.52)	2.34* (1.27, 4.33)	2.87** (2.24, 3.68)
Secondary (ref: tertiary)	0.87 (0.60, 1.25)	1.39 (0.76, 2.55)	1.95** (1.48, 2.56)	1.31 (0.76, 2.27)	1.13 (0.59, 2.15)	1.59** (1.27, 1.99)
Rural residence	2.29** (1.73, 3.04)	1.13 (0.84, 1.54)	0.94 (0.80, 1.11)	1.74** (1.31, 2.29)	1.25 (0.91, 1.71)	1.00 (0.87, 1.16)
Marital status						
Never	1.56* (1.11, 2.20)	2.26** (1.66, 3.07)	2.12** (1.65, 2.71)	1.46 (0.99, 2.17)	10.39** (6.82, 15.84)	2.34** (1.89, 2.90)
Formerly (ref: currently married/in union)	0.82 (0.57, 1.17)	1.31 (0.88, 1.93)	1.06 (0.80, 1.41)	1.18 (0.80, 1.74)	1.82 (0.98, 3.36)	1.52** (1.20, 1.92)
Has no children	0.98 (0.78, 1.25)	2.41** (1.80, 3.22)	1.36* (1.05, 1.75)	1.55* (1.05, 2.29)	3.59** (2.34, 5.49)	2.04** (1.66, 2.49)
Unemployed	2.23* (1.15, 4.35)	1.15 (0.90, 1.46)	1.14 (0.96, 1.36)	1.16 (0.94, 1.44)	2.15** (1.41, 3.28)	1.03 (0.88, 1.19)
No health insurance	2.11** (1.59, 2.81)	1.70 (0.73, 3.98)	1.34* (1.04, 1.73)	3.29* (1.04, 10.42)	‐	1.06 (0.76, 1.48)
One lifetime sex partner (ref: 2 or more)	1.38** (1.20, 1.60)	1.41** (1.17, 1.69)	1.47** (1.24, 1.75)	1.03 (0.73, 1.43)	1.27* (1.03, 1.57)	1.43** (1.25, 1.63)
Away from home ≥1 month per year (ref: <1 month)	0.46** (0.39, 0.54)	1.05 (0.90, 1.23)	1.01 (0.88, 1.17)	1.08 (0.89, 1.32)	0.85 (0.67, 1.06)	0.83** (0.75, 0.93)
Not circumcised	1.00 (0.73, 1.38)	1.42** (1.19, 1.70)	3.07** (2.20, 4.29)	1.93* (1.53, 2.45)	2.29** (1.71, 3.05)	1.63** (1.42, 1.87)

*p <* *0.05; **p <* *0.001; ref: reference category.

### 3.4 Survey testing and HIV positivity among participants who had never tested previously

**Figure 2:**
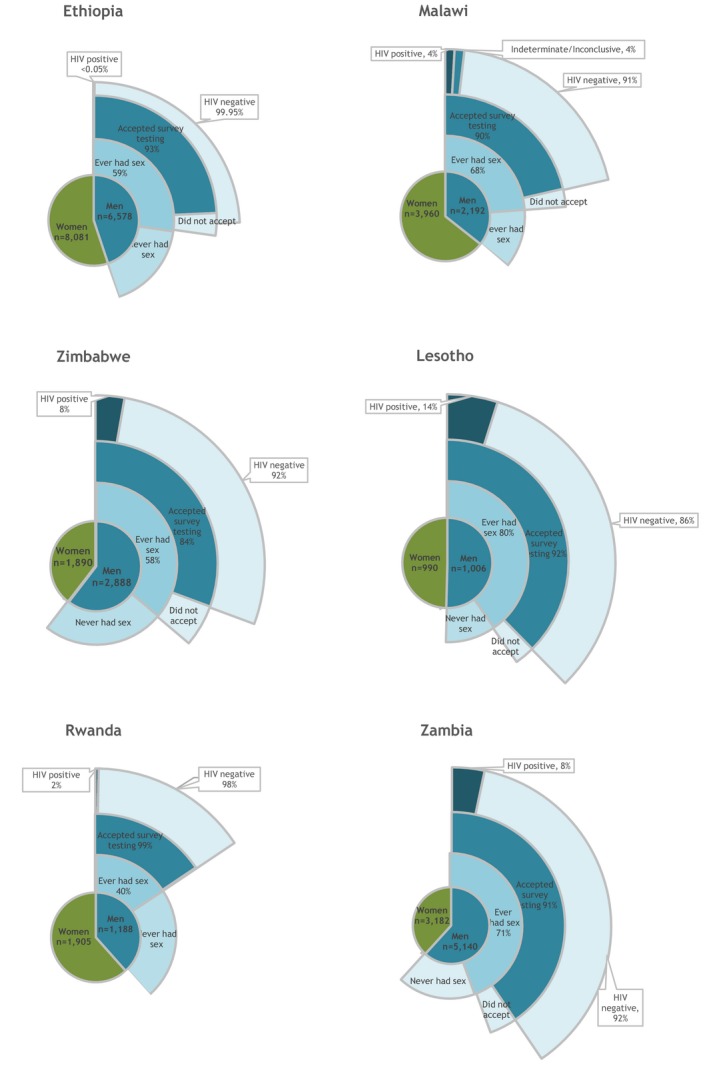
Survey HIV testing results of men who had ever had sex and never tested before.

High proportions of never‐tested men accepted HIV testing during the surveys (93% in Ethiopia, 90% in Malawi, 85% in Zimbabwe, 99% in Rwanda, 93% in Lesotho and 91% in Zambia) (Figure 2). HIV positivity varied widely (<0.05% to 14%) among those who tested for HIV for the first time: <0.05% of men who had ever had sex were HIV positive in Ethiopia, 4% in Malawi, 8% in Zimbabwe, 14% in Lesotho, 2% in Rwanda and 8% in Zambia (Table 2). In most countries, never‐tested men had lower HIV positivity than previously tested men, apart from Rwanda where men aged 30 to 34 years had significantly higher HIV positivity than men who had previously tested. Across all included countries, HIV positivity was highest in older men over 35 years old regardless of testing history.

**Table 2:** HIV‐positive tests among sexually active men by age and prior testing

Age (yrs)	Ethiopia 2016 (N = 12,688)		Malawi 2015 (N = 7478)		Zimbabwe 2015 (N = 8396)		Lesotho 2014 (N = 2931)		Rwanda 2014 (N = 6217)		Zambia 2013 (N = 14,765)	
	Ever tested, n (%)	Never tested, n (%)	Ever tested, n (%)	Never tested, n (%)	Ever tested n (%)	Never tested, n (%)	Ever tested, n (%)	Never tested, n (%)	Ever tested, n (%)	Never tested, n (%)	Ever tested, n (%)	Never tested, n (%)
15 to 19	0 (0)	1 (0.07)	9 (1.58)	2 (0.33)*	5 (1.94)	2 (0.34)*	6 (1.64)	6 (5.32)	0 (0)	0 (0)	29 (3.71)	32 (3.21)
20 to 24	2 (0.63)	1 (0.01)**	9 (1.34)	4 (0.83)	27 (4.25)	9 (2.49)	21 (9.21)	8 (5.30)	9 (1.81)	0 (0)	100 (7.56)	31 (5.79)
25 to 29	10 (0.58)	4 (0.02)**	48 (6.86)	8 (4.15)	62 (8.41)	13 (4.86)	51 (18.89)	14 (15.66)	14 (1.67)	1 (1.92)	142 (10.00)	40 (10.19)
30 to 34	7 (0.52)	4 (0.65)	70 (8.92)	8 (12.24)	109 (13.74)	29 (12.36)	68 (29.43)	19 (23.32)	14 (1.84)	4 (9.54)*	198 (14.97)	41 (10.15)
35 to 39	18 (1.46)	1 (0.01)**	83 (11.88)	11 (12.37)	132 (19.47)	29 (13.21)	78 (41.14)	19 (40.12)	21 (3.39)	0 (0)	231 (18.83)	36 (11.52)*
40 to 44	26 (2.49)	4 (0.71)	68 (14.55)	7 (11.65)	170 (31.41)	16 (10.67)**	67 (45.78)	11 (34.97)	18 (3.97)	0 (0)	208 (22.26)	38 (15.70)
45 to 49	14 (2.97)	1 (0.16)**	64 (20.38)	12 (15.14)	115 (26.47)	20 (15.20)*	39 (33.65)	8 (20.67)	36 (9.71)	1 (3.37)	136 (21.35)	28 (13.31)*
50 to 54	12 (1.48)	4 (1.01)	58 (24.12)	3 (6.10)*	82 (30.85)	14 (22.19)	44 (33.05)	6 (21.13)	20 (6.04)	1 (2.54)	102 (22.81)	19 (12.16)*
55 to 59	2 (1.20)	1 (0.05)*	‐	‐	‐	‐	31 (29.17)	6 (17.36)	11 (4.49)	3 (3.78)	60 (20.11)	10 (4.92)**
Total	91 (1.27)	21 (0.32)*	409 (9.13)	55 (4.49)**	702 (15.84)	132 (7.80)**	405 (24.30)	97 (14.08)**	143 (3.30)	10 (1.76)	1206 (14.67)	275 (8.35)**

Survey results are weighted.

*p < 0.05; **p < 0.001.

## 4 Discussion

A gender gap in HIV testing persists in sub‐Saharan Africa. We found that compared to women, men in six sub‐Saharan African countries were approximately twice as likely to have never been tested for HIV. Being single, having lower levels of education and not having any children were associated with higher odds for not having tested for HIV among men, a finding consistent with a 2012 analysis of DHS data 35 and two population‐based surveys in Tanzania and Zambia which found that being younger and having less education are associated with never previously testing for HIV 24, 25. In our findings, men who had never previously tested were unemployed or employed in agriculture, household or domestic services, had less wealth, and lower health insurance coverage, pointing to an inequity in access to HIV testing services for individuals with lower incomes. Men who had never been tested for HIV exhibited sexual risk factors for HIV, and thus, are important to reach with testing. Despite never testing before, high proportions of men accepted testing during the surveys (85% to 99%), and although HIV positivity was high in some settings, it was lower than among those who had tested previously. HIV positivity of never tested men was highest among men in older age groups, indicating a need for increased coverage of HIV testing among older men to identify those living with undiagnosed HIV.

Male gender norms in sub‐Saharan Africa may accentuate risk‐taking and discourage health‐seeking behaviour 16. A DHS analysis focusing on men's knowledge and psychosocial characteristics found that men who were accepting of gender‐based violence, demonstrated low HIV knowledge or held stigmatizing attitudes related to HIV were more likely to report never testing for HIV 22. Qualitative research shows that some reasons men may not test for HIV are fear of HIV stigma 36, 37, strong gender‐based social roles 16, 37 employment which makes it inconvenient to reaching health clinics during opening hours 16, 37 and the perception that testing facilities are not male‐friendly because they are dominated by female staff and patients and do not offer services that are focused on men's general health and well‐being 36, 38. HIV testing services (HTS) will need to adapt how and where services are provided to reach more men. Adolescent males who belong to key populations are especially at risk of acquiring HIV 39, and their preferences for testing should also be considered.

Men who had never previously been tested accepted HIV testing during the DHS at a much higher rate than global HIV testing uptake for men (85% to 99% vs. 30%) 40. This high uptake indicates that men who have never tested before are willing to test for HIV, but may not have been offered an easy opportunity for testing. Presently, men do not have access to the same opportunities to test for HIV as women who access health services more often 41 and are regularly offered HIV testing during antenatal care. Other studies have found similar results indicating willingness to test among men. One analysis of community‐based testing offered through a population‐based survey in Tanzania found that half of men in the survey had never previously tested for HIV and first‐time male testers were associated with testing HIV positive 25. Another population‐based survey in Zambia found that 57% of never tested men accepted home‐based HIV testing when offered 24. Willingness to test can also be related to the subtleties of how HIV testing is offered. An opt‐out HTS approach, as in the survey setting of the DHS, yields significantly higher HIV testing acceptance rates 42. Thus, the way men are approached with the question of whether to uptake HIV testing can have a substantial effect on their decision to test.

Our findings of higher rates of circumcision among men who had previously tested for HIV could indicate that HIV testing provided alongside other services is one way to make it easier for men to test. High HIV testing uptake within Tuberculosis (TB) programmes is a further example of men accepting testing when HTS is combined with other health services. Men make up a large majority of TB cases globally and, in 2017, 86% of TB patients in the African region reported knowing their HIV status 43. Higher uptake of HTS when it is offered alongside other services points to a willingness to test among people when HIV testing is routinely and easily accessible. Therefore, our findings of lower HIV testing rates among young, never married men with lower levels of education and without children could primarily reflect the lack of access to HTS and high opportunity costs of facility‐based testing for men.

HIV positivity in DHS survey testing ranged from <1% to 14% among men who never tested and was lower than for men who had previously tested. A comparative analysis of DHS survey results in 15 African countries found a majority of people living with HIV living in rural areas in nine countries, including Lesotho, Malawi, Rwanda and Zimbabwe. The report also shows that urban residence is a factor in prior testing among HIV‐positive men 23. Combined with our findings that men who never previously tested were more likely to reside in rural areas, this indicates an opportunity for HIV case finding in these locations. In our analysis, HIV seropositivity among men who had never tested was lower than the national prevalence for men in all countries, consistent with trends in high burden countries with established HIV testing programmes, which show that finding and testing the remaining men with undiagnosed HIV will become more laborious and costly as the prevalence of undiagnosed HIV in the population diminishes, especially when undiagnosed men may live in rural locations which are difficult to reach with existing services. When analysed by age group, HIV positivity was higher in older men who never previously tested compared to younger men who never tested, consistent with age‐specific HIV incidence and prevalence in many African countries which is markedly lower among younger men, and increases with age to become higher among middle‐aged and older men 44, 45, 46, 47, 48. Countries will need to prioritize approaches which target those at higher risk, thus reaching older men who are first‐time testers with acceptable HIV testing programmes will be imperative.

New testing approaches must consider men's preferences, context, epidemiology and available resources 3, 49. A study in Kenya showed that men preferred to test outside of facilities 50. Recent studies indicate that community‐based testing strategies can reach > 90% testing coverage 51 and men with undiagnosed HIV can be reached with special community‐based efforts 52. Some outreach to men has succeeded through door‐to‐door HTS 53, home‐based couples testing 54, workplace programmes 55, 56, mobile testing services 25, 49, 57, 58, 59, social network interventions 60 and incentives to test 59, but existing implementation has not been extensive. WHO recommendations on HIV self‐testing (HIVST) and partner notification in 2016 61, offer two new approaches to reach men. HIVST is a convenient and private testing strategy that has been shown to be both acceptable and effective in case finding among men. According to a meta‐analysis, HIVST doubled HIV testing uptake in men in three randomized controlled trials in Hong Kong SAR and Kenya 62. Compared with standard HIV testing services, HIVST was also found to double the likelihood of an HIV‐positive diagnosis 62. Voluntary assisted partner notification has also demonstrated success in increasing uptake of HIV testing 63, including reaching first‐time testers 63 and those with previously undiagnosed HIV infection 64, 65. A meta‐analysis of partner notification in four randomised controlled trials found 20% to 72% of partners tested HIV positive using this approach 63. A mixed methods partner notification study in Tanzania found that male index clients reported a greater number of partners and successfully referred more partners to HIV testing than female index clients 66, showing that men can be successfully engaged in HTS and partner notification.

A limitation of our analysis is that the surveys rely on self‐reported HIV testing history and sexual behavioural characteristics, which can be limited by response bias and social desirability bias, especially given the social stigma of HIV that persists in sub‐Saharan Africa. A study that investigated self‐reporting of HIV testing history in population‐based surveys in Tanzania showed that previously tested individuals are more likely to misreport never having tested if they have no primary school education, have never been married and reside in a remote rural area 67. Thus, the association in the DHS data between never having tested for HIV and factors such as low educational attainment, never being married and rural residence could be due in part to differential misreporting of HIV testing history. However, the patterns we observed for self‐reported HIV testing history were consistent across countries, except in Ethiopia and Rwanda where HIV prevalence is low and different dynamics of the epidemic are expected. HIV test results were not returned to participants in the surveys, a practice which has been discouraged 3, 68, 69. Not receiving test results may have reduced fears related to testing HIV positive, impacting our findings of high uptake of testing among men, and therefore may not be generalizable to programmatic settings where participants learn their serostatus after testing. However, we were able to compare testing acceptance rates to other recent population‐based surveys which report HIV status to participants and link them to treatment, and found that rates of acceptance of HIV testing were similar.

## 5 Conclusions

A substantial differential exists between men and women who have never tested for HIV in most of the countries in this study. Although higher proportions of men than women had never tested for HIV, between 84% and 99% of men did test when offered during a survey. Finding opportunities to offer HIV testing specifically to single men with no children, older men who have never tested, and those disadvantaged with less schooling and employment, alongside other facility and community‐based services, will be important in identifying those living with undiagnosed HIV. Attention to the uptake of HIV testing, treatment and prevention in men is required for men's health and well‐being, and is critical for epidemic control. Findings from implementation research should be used to inform the design of male‐friendly HIV testing and prevention programmes.

## Competing interests

Authors declare no conflicts of interest.

## Authors’ contributions

SD conceptualized the research question and designed the study with CQ. CQ and DTK performed the data analysis. CQ drafted the manuscript. CQ, DTK, CCJ, RB and SD contributed intellectual content, provided revisions and approved of the final manuscript.

## Abbreviations

aOR: adjusted odds ratio
ART: antiretroviral treatment
CD4: cluster of differentiation 4
DHS: Demographic and Health Surveys
HIV: human immodeficiency virus
HIVST: human immodeficiency virus self‐testing
HTS: HIV testing services
IQR: interquartile range
IRB: institutional review board
TB: tuberculosis
WHO: World Health Organization

## Supporting information


**Table S1.** Characteristics of male survey respondents by HIV testing history. ANC antenatal care.
