# Supplementary material for: Who are the missing men? Characterising men who never tested for HIV from population‐based surveys in six sub‐Saharan African countries
Source: J Int AIDS Soc. 2019 Oct 20;22(10):e25398. doi: 10.1002/jia2.25398 (PMC6801221; doi:10.1002/jia2.25398)
Supplement: Supplementary file 1 — Table S1. Characteristics of male survey respondents by HIV testing history. ANC antenatal care. [file JIA2-22-e25398-s001.docx]

3449 (57)

**Characteristics of male survey respondents by HIV testing history**

**Ethiopia (2016)**

**Malawi (2015-16)**

**Zimbabwe (2015)**

**Lesotho (2014)**

**Rwanda (2014-15)**

**Zambia (2013-14)**

**---------(N=12,688)---------**

**----------(N=7,478)----------**

**-----------(N=8,396)----------**

**----------(N=2,931)----------**

**----------(N=6,214)----------**

**-----------(N=14,763)-----------**

**Characteristics**

**Never tested**

**Tested**

**Never tested**

**Tested**

**Never tested**

**Tested**

**Never tested**

**Tested**

**Never tested**

**Tested**

**Never tested**

**Tested**

**n=6,578**

**n=6,110**

**n=2,192**

**n=5,286**

**n=2,889**

**n=5,507**

**n=1,006**

**n=1,925**

**n=1,188**

**n=5,026**

**n=5,145**

**n=9,618**

n (%)

n (%)

n (%)

n (%)

n (%)

n (%)

n (%)

n (%)

n (%)

n (%)

n (%)

n (%)

**Age:**

15-19

1957 (30)

576 (9)

**

1139 (53)

707 (12)

**

1261 (45)

804 (15)

**

372 (37)

318 (17)

**

657 (56)

624 (12)

**

2226 (43)

1118 (11)

20-24

993 (15)

976 (15)

396 (17)

984 (20)

493 (17)

883 (15)

219 (23)

315 (17)

224 (19)

775 (15)

764 (15)

1542 (16)

25-29

767 (12)

1263 (20)

136 (7)

894 (17)

285 (10)

881 (19)

107 (11)

287 (16)

81 (7)

883 (17)

429 (9)

1505 (16)

30-34

650 (10)

935 (16)

118 (5)

827 (16)

247 (8)

857 (16)

89 (9)

256 (13)

46 (4)

886 (18)

396 (8)

1498 (16)

35-39

604 (10)

771 (12)

131 (6)

735 (14)

201 (7)

731 (13)

54 (6)

221 (11)

16 (1)

543 (11)

328 (6)

1343 (14)

40-44

583 (8)

634 (11)

101 (5)

518 (10)

168 (6)

629 (12)

50 (5)

172 (9)

28 (2)

441 (9)

323 (6)

1064 (11)

45-49

437 (7)

432 (8)

96 (4)

356 (7)

152 (6)

426 (8)

41 (4)

125 (7)

23 (2)

358 (7)

277 (5)

717 (7)

50-54

319 (4)

304 (5)

75 (4)

265 (5)

82 (3)

296 (5)

37 (3)

129 (6)

46 (4)

303 (6)

217 (4)

503 (5)

55-59

268 (4)

219 (4)

-

-

-

37 (3)

102 (5)

67 (6)

216 (4)

188 (3)

335 (4)

**Education:**

None

2329 (35)

1188 (25)

**

122 (6)

277 (6)

**

26 (1)

31 (1)

**

124 (11)

205 (9)

**

99 (8)

560 (12)

**

228 (4)

326 (4)

Primary

3066 (52)

2265 (40)

1481 (69)

2771 (54)

876 (31)

979 (19)

559 (54)

814 (41)

820 (70)

3151 (64)

2436 (47)

3545 (37)

Secondary

823 (10)

1410 (20)

548 (22)

1928 (34)

1839 (63)

3685 (67)

296 (32)

728 (40)

237 (19)

1045 (20)

2278 (45)

4745 (50)

Higher than secondary

360 (3)

1247 (15)

41 (2)

310 (6)

148 (5)

812 (14)

27 (4)

178 (10)

32 (2)

273 (5)

199 (4)

1006 (10)

**Marital status:**

Ever in a union

3500 (53)

4083 (71)

**

691 (32)

3855 (74)

**

1038 (35)

3739 (69)

**

333 (32)

1,121 (57)

**

175 (15)

3295 (66)

**

1976 (38)

6873 (71)

Never in a union

3078 (47)

2027 (29)

1501 (68)

1431 (26)

1851 (65)

1768 (31)

673 (68)

804 (43)

1013 (85)

1734 (34)

3172 (62)

2752 (29)

**Residence:**

Rural

5281 (88)

3541 (71)

**

1760 (84)

4057 (80)

**

1898 (69)

3042 (61)

**

800 (77)

1,228 (61)

**

257 (16)

1350 (21)

**

2970 (56)

4975 (53)

Urban

1297 (12)

2569 (29)

432 (16)

1229 (20)

991 (31)

2465 (39)

206 (23)

697 (39)

931 (84)

3679 (79)

2178 (44)

4650 (47)

**Employed:**

Yes

5507 (90)

5591 (95)

**

1613 (76)

4776 (91)

**

1836 (63)

4386 (80)

**

586 (61)

1375 (73)

**

890 (75)

4568 (91)

**

3646 (71)

8373 (87)

No

1071 (10)

519 (5)

579 (24)

510 (9)

1050 (37)

1113 (20)

420 (39)

551 (27)

293 (25)

458 (9)

1501 (29)

1251 (13)

**Occupation:**

Professional

226 (2)

694 (12)

**

62 (4)

434 (9)

**

128 (6)

640 (15)

**

15 (3)

101 (6)

**

18 (2)

279 (6)

**

129 (3)

622 (7)

Clerical

33 (<1)

100 (2)

26 (2)

122 (2)

10 (<1)

65 (1)

10 (1)

45 (3)

2 (<1)

23 (<1)

13 (<0.5)

111 (1)

Sales

343 (5)

526 (9)

45 (3)

162 (3)

117 (6)

315 (7)

29 (5)

112 (9)

71 (8)

344 (7)

450 (14)

1168 (15)

Agriculture (self-employed)

-

-

875 (51)

2002 (40)

350 (23)

631 (17)

311 (49)

354 (23)

380 (44)

2122 (48)

702 (18)

1357 (16)

Agriculture (employed)

3888 (81)

2642 (47)

15 (1)

61 (2)

173 (9)

204 (5)

13 (3)

37 (3)

156 (18)

532 (12)

1500 (39)

2608 (31)

Household and domestic

-

-

29 (2)

77 (1)

91 (5)

136 (3)

9 (2)

11 (1)

58 (5)

110 (2)

-

Services

101 (<1)

261 (5)

20 (1)

123 (3)

345 (17)

811 (17)

32 (5)

163 (13)

19 (2)

167 (3)

108 (3)

334 (4)

Skilled manual

361 (4)

852 (15)

167 (10)

669 (15)

480 (26)

1232 (28)

93 (18)

297 (22)

73 (8)

598 (13)

464 (13)

1202 (15)

Unskilled manual

134 (2)

210 (4)

374 (27)

1126 (25)

46 (2)

131 (3)

48 (10)

156 (13)

112 (13)

389 (9)

109 (4)

465 (6)

Other

421 (6)

306 (5)

-

-

96 (6)

221 (5)

25 (4)

89 (7)

-

-

145 (5)

446 (6)

**Wealth:**

Lowest

2109 (21)

799 (9)

**

320 (17)

727 (16)

*

479 (17)

697 (14)

**

243 (19)

285 (12)

**

171 (15)

727 (15)

884 (16)

1408 (15)

Low

1092 (21)

718 (15)

400 (19)

918 (18)

529 (20)

824 (17)

207 (20)

362 (17)

202 (17)

867 (18)

1054 (18)

1824 (18)

Middle

1014 (22)

724 (16)

454 (22)

989 (19)

572 (22)

908 (18)

246 (24)

364 (19)

215 (19)

964 (20)

1097 (20)

2005 (18)

High

949 (20)

976 (23)

463 (20)

1108 (21)

668 (21)

1399 (24)

175 (19)

420 (24)

289 (25)

1069 (22)

1098 (24)

2143 (23)

Highest

1414 (16)

2892 (36)

555 (22)

1544 (26)

641 (21)

1679 (27)

135 (17)

494 (28)

311 (24)

1402 (25)

1015 (23)

2245 (26)

**Mobility:**

High mobility (1+ trips)

2373 (35)

**

778 (37)

2362 (45)

**

1481 (51)

3392 (62)

**

457 (49)

1022 (53)

*

371 (30)

2008 (39)

**

1831 (34)

4630 (46)

Low mobility (0 trips)

4205 (65)

2661 (43)

1414 (63)

2924 (55)

1408 (49)

2115 (38)

549 (51)

901 (47)

816 (70)

3015 (61)

3308 (66)

4985 (54)

**Have children:**

Yes

3236 (49)

3579 (63)

**

637 (29)

3701 (71)

**

945 (32)

3491 (64)

**

295 (28)

1041 (53)

**

173 (15)

3229 (65)

**

1934 (37)

6876 (71)

No

3342 (51)

2531 (37)

1555 (71)

1585 (29)

1944 (68)

2016 (36)

711 (72)

884 (47)

1015 (85)

1800 (35)

3214 (63)

2749 (29)

**Health insurance:**

Yes

245 (5)

484 (9)

**

20 (1)

152 (3)

**

230 (7)

901 (15)

**

5 (1)

52 (3)

**

-

-

79 (2)

333 (3)

No

6333 (95)

5626 (91)

2172 (99)

5134 (97)

2659 (93)

4606 (85)

1001 (99)

1873 (97)

-

-

5066 (98)

9285 (97)

**Ever had sex:**

Yes

3993 (59)

5077 (83)

**

1465 (68)

4928 (94)

**

1728 (58)

4824 (87)

**

804 (80)

1781 (92)

**

486 (40)

4141 (83)

**

3700 (71)

9032 (94)

No

2585 (41)

1033 (17)

727 (32)

358 (6)

1160 (42)

682 (13)

202 (20)

144 (8)

702 (60)

885 (17)

1443 (29)

585 (6)

*****

<0.05

******

<0.001

Note: Survey results are weighted.
